# Exposure of Farmers and Spouses to Glyphosate in Morocco: Urinary Levels and Predictors of Exposure

**DOI:** 10.3390/toxics14050381

**Published:** 2026-04-29

**Authors:** Imane Berni, Aziza Menouni, Matteo Creta, Kaoutar Chbihi, Hala Chetouani, Said Abou-Said, Lode Godderis, Samir El Jaafari, Radu-Corneliu Duca

**Affiliations:** 1 Laboratory of Cell Biology and Molecular Genetics, Department of Biology, Faculty of Sciences, Ibn Zohr University, Agadir 80000, Morocco; 2 High Institute of Nursing Professions and Technical Health, Agadir 80000, Morocco; 3 Human Epidemiology and Environmental Health Team, Faculty of Sciences, Moulay Ismail University, Meknes 50000, Morocco; 4 Environment and Health Unit, Department of Public Health and Primary Care, Katholieke Universiteit Leuven, 3000 Leuven, Belgium; 5 Unit Environmental Hygiene and Human Biological Monitoring, Department of Health Protection, National Health Laboratory (LNS), 3401 Dudelange, Luxembourg; 6 IDEWE, External Service for Prevention and Protection at Work, 3001 Heverlee, Belgium; 7 Department Health Protection, Health Directorate, 8008 Strassen, Luxembourg; radu.duca@ms.etat.lu

**Keywords:** biological monitoring, urine, farmers, health risk

## Abstract

Levels of glyphosate and their metabolite AMPA were measured in urine of farmers and their spouses that lived in intensively farmed area in Morocco. The levels were used as proxies to determine the exposure of these target population to herbicides. Determinants of exposure to glyphosate were also assessed. Urine was collected from 104 farmers, 50 spouses and 50 people from the general adult population and information from sociodemographic characteristics and occupational exposure was collected from questionnaires administrated to farmers and spouses. The detection frequency was 67% for glyphosate and 62% for AMPA among farmers, and 56% for glyphosate and 48% for AMPA among spouses, with the mean concentration of 1.22 μg L^−1^ and 0.85 μg L^−1^ among farmers, and 0.58 μg L^−1^ and 0.50 μg L^−1^ among spouses, respectively. Nevertheless, mean values of glyphosate and AMPA for general population were below the reported quantification limits. Multiple regression analysis showed that smoking status, applying glyphosate in the last 7 days, and glove use are the most important contribution to urinary levels of glyphosate and AMPA among farmers, and proximity of home to spraying area, and herbicides drift enters house are the main predictors of urinary glyphosate and AMPA exposure among spouses. The GMs of estimated daily intake were 1.26 and 1.39 µg/kg BW/day for glyphosate and AMPA among farmers, and 0.56 and 0.96 µg/kg BW/day for glyphosate and AMPA among spouses, respectively. This study provides further evidence on factors associated with glyphosate and AMPA exposure, especially in developing countries.

## 1. Introduction

Glyphosate (N-(phosphonomethyl)-glycine) is regarded as the most extensively used herbicide globally since its introduction into agricultural practice in the 1970s, with approximatively 750 formulations containing the active ingredient [1,2]. Over 8 million metric tons of glyphosate have been used globally in recent decades [3,4]. Glyphosate is extensively used to preserve cereals, orchids and vegetables crops, as well as in urban areas, roads and train tracks, garden and home against weeds, due to its wide range of action [5]. Glyphosate has recently attracted international attention due to its designation as ‘Group 2A—probably carcinogenic to humans’ by the International Agency for Research on Cancer (IARC), due to its unequivocal proof of carcinogenicity in experimental animals [6]. Despite the IARC findings, the European Food Safety Authority (EFSA) found that there was insufficient scientific evidence to categorize glyphosate as possibly carcinogenic to humans, and established an acceptable daily intake (ADI) value of 0.5 mg/kg BW/day [7]. IARC also reported “high” evidence of genotoxicity in either pure glyphosate or glyphosate-based formulations [8]. Human DNA and chromosomes were shown to be damaged, although the mutagenic consequences were not observed in bacteria [6]. Dysfunctions in DNA methylation can activate oncogenes, while hypermethylation can suppress the genes responsible for stopping the tumors [9]. Glyphosate and AMPA can therefore alter the balance between cell proliferation and programmed cell death, and disrupt normal neurotransmission [10].

After application, glyphosate enters the environment (water, soil, and plants) and is degraded by microorganisms into its metabolite aminomethylphosphonic acid (AMPA) [11,12]. AMPA is another source of toxicity and has a similar toxicological profile to glyphosate; it has been assigned the same ADI as glyphosate [7]. In recent years, several studies have reported the presence of glyphosate and AMPA in different matrices such as food [13], groundwater and surface water [14], and ambient air [15]. The presence of AMPA in urine is primarily due to its direct ingestion through food and water, and to a lesser extent to human glyphosate metabolism [16].

Sufficient evidence confirms that exposure to glyphosate in the general population has been linked to a variety of health issues including kidney damage, different cancers, reproductive system and neurological and emotional conditions such as autism, anxiety, Alzheimer’s and Parkinson’s disease [16,17]. Furthermore, occupational exposure to glyphosate generally occurs through skin absorption, inhalation, and ingestion [18,19]. Some studies showed that medium-high level exposure to glyphosate might increase the risk of follicular lymphoma, non-Hodgkin lymphoma [20,21], and chronic lymphocytic leukemia [22].

Data from human research suggest that absorbed glyphosate is generally not metabolized and rapidly excreted unchanged in urine [23]. According to this study, the total dose recovered as unchanged in the urine samples is around 1–6%, and the excretion half-life is between 6 and 10 h [24,25,26]. Likewise, refs. [25,27] estimates a human half-life of glyphosate of 9 h and between 5.5 and 10 h, respectively. For AMPA, according with a recent oral study conducted by [23], only 0.01–0.04% of glyphosate is metabolized to AMPA.

For biomonitoring studies, urinary glyphosate is a suitable biomarker for glyphosate exposure. Similarly, AMPA in urine serves as a biomarker for both glyphosate that is transformed to AMPA and direct exposure to the metabolite AMPA. There have been limited biomonitoring studies of glyphosate covering occupational exposure. In a pilot study conducted by Irish horticulturists, 17 urine samples were analyzed, of which glyphosate were quantified with average level of 0.7 μg/L [28]. In a recent study, the levels of glyphosate and AMPA in urine of Chinese workers (*n* = 134) were measured. In 86% and 81% of the samples, glyphosate and AMPA were detected with median values of 292 μg/L and 68 μg/L, respectively [29]. In order to strengthen epidemiological research, encourage the safe and suitable use of glyphosate, and support regulatory exposure assessment, reliable data on the extent of occupational glyphosate exposure are needed across different countries.

While many studies have investigated potential exposure among farmers and agricultural workers, far fewer have examined exposure among farm families as a result of household contamination. To consider some of the above-mentioned gaps in the current literature on glyphosate human biomonitoring, the objectives of this study were: (i) to determine the urinary concentrations of glyphosate and AMPA among farmers, spouses and the general population in the Saiss plain in Morocco; (ii) to test associations between urinary levels of the reported data and sociodemographic characteristics and occupational exposure to identify possible sources of exposure and (iii) to perform a risk assessment for glyphosate and AMPA through urine biomonitoring.

## 2. Materials and Methods

### 2.1. Standards and Reagents

All chemicals were of analytical grade, unless otherwise stated. The reference compounds (glyphosate and AMPA) were obtained from Sigma Aldrich (Belgium). For Novum Simplified Liquid Extraction (SLE) 12cc tube, and chemical analysis, gradient grade methanol (MeOH), dichloromethane (DCM), acetonitrile (ACN), and formic acid (FA) were purchased from Sigma-Aldrich (Diegem, Belgium). Ultrapure water was produced using a Milli-Q gradient water system (Millipore, Billerica, MA, USA).

### 2.2. Study Population

This study is part of PAPOE study (Parental Pesticides and Offspring Epigenome), a cross-sectional study assessing the occupational exposure to pesticides among farmworkers in North-central Morocco. Details of the PAPOE study design and data-collection procedures have been described elsewhere [30] and ethics can be found in [31]. Briefly, farmers and spouses were eligible for participation if they met the following criteria: (i) participants aged > 18 years; (ii) both farmers and spouses had to be in good health and had never been diagnosed with renal disease; (iii) a minimum 10 year residency in Saiss plain and are official or seasonal farmworkers, (iv) spouses should not be pregnant at the time of urine collection. A control group of sex-and-age-matched individuals were also recruited at the same time and included healthy adults living in our study area whose jobs were not linked to agriculture and did not employ the use of herbicides. Participants took part in the study on a voluntary basis.

### 2.3. Participants, Sample and Data Collection

Participants’ recruitment was organized and collected in late spring to early summer between May and June 2019 during the growing season [31]. Participants collected their first morning urine in a clean 100 mL polypropylene container. Collection bottles were stored in a refrigerator or in cool bags with cooling pads for transportation to the Health and Environment Laboratory of Moulay Ismail University (Morocco) and stored at −80 °C. Urine samples were shipped to the Health and Environment Laboratory of Leuven, Belgium for analysis [31].

Prior to urine collection, a written questionnaire administered by qualified staff was filled in by each participant providing self-reported socio-demographic information. Collected data included age, education level (none, primary, secondary, university degree), marital status (married, divorced, widowed or single), and smoking status.

In order to collect data on farmers’ exposure to environmental occupational herbicides, the questionnaire also included the following: exposed to herbicides during last year, days per year mixing/applying herbicides, applied glyphosate in last 7 days before study, personal protective equipment (PPE) use, reuse of PPE, take a break during the task, task the worker completed (mixing/loading, spraying the chemical, clinging the equipment, collection of the herbicide from the store, storage of herbicides in house). Residency environment exposure questions for spouses included: spouses mixed any pesticides in the week before study, time with the windows open during the day, garden or plants at home, proximity of home to spraying area, and reuse of empty containers.

### 2.4. Laboratory Analysis

#### 2.4.1. Urine Sample Chemical Analysis

All samples were prepared and analyzed for glyphosate and AMPA following the previously described analytical method [31]. In brief, sample clean-up was done by passing the 1 mL of urine through a Novum SLE cartridge, preconditioned with 1 mL of 3% aqueous formic acid. Once the sample was completely soaked in the sorbent, it was left to stand for 5 min. We applied 9 mL of DCM and ACN (8/2) (3 × 3) elution solvent to the sorbent and allowed it to completely elute from the sorbent via gravity. The extracts were evaporated to dryness and transferred into injection vials in 200 μL of MeOH and HPLC water (2/8) [31].

#### 2.4.2. Instrumental Analysis

Analysis was performed using the UPLC-MS/MS method interfaced with an electrospray ionization source in negative mode (ES−); mass spectrometer parameters used are included in previous paper [31]. The LOD and LOQ of the analytical method were determined from calibration standards. For all compounds, the LOQ and LOD were determined using the lowest concentration level with acceptable accuracy and precision. The LOQ was 0.7 μg L^−1^ for glyphosate and 1 μg L^−1^ for AMPA, whereas the LOD was 0.1 μg L^−1^ for glyphosate and 0.5 μg L^−1^ for AMPA. Analytical reproductivity was frequently below 20% and recoveries ranged between 80 et 120% for both glyphosate and AMPA.

### 2.5. Sample Size and Statistical Analysis

A total of 130 farmers and 64 spouses were initially recruited. Among them, urine samples of sufficient volume (1 mL) and quality were available for 104 farmers and 50 spouses and were therefore included in the analysis. In addition, 50 adults from the general population were recruited as a comparison group. Overall, the study comprised three independent groups: farmers (*n* = 104), spouses of farmers (*n* = 50), and individuals from the general population (*n* = 50). The sample size was determined by participant availability within an ongoing environmental monitoring study conducted in the Saiss plain. Consequently, the sample size was based on feasibility considerations rather than on an a priori statistical power calculation.

Given the non-normal distribution of urinary glyphosate and AMPA concentrations, group comparisons were performed using the Wilcoxon rank-sum test. Although the sample size was primarily determined by feasibility, a post hoc power assessment indicated that the total sample size (*n* = 204), at a significance level of α = 0.05, was sufficient to detect moderate between-group differences. Biomonitoring results of glyphosate and AMPA as well as demographic characteristics data and reported herbicide exposure are reported as frequency tables, calculated means, standard deviations, percentiles, 95 confidence intervals, etc. Normality of glyphosate and AMPA concentrations was assessed using box-and-whisker plots. Log transformation of non-normally distributed data did not improve normality; therefore, untransformed data were used. Data below the respective detection limit were replaced by LoD/2 for statistical analysis. Bivariate analyses were then conducted to examine the association between urinary compounds, and reported herbicide exposure, socio-economic factors, and demographic factors. Tests applied in bivariate analysis for two continuous variables included simple linear regression analysis, rank-sum correlations (for data not normally distributed) or Pearson correlation coefficient (for normally distributed data) and *t*-test (for normally distributed data) or Wilcoxon rank sum test (for data not normally distributed) was used for one dichotomous and one continuous variable, and Chi-Square testing for two dichotomous variables. Reported glyphosate and AMPA exposures, socio-economic parameters, and demographic determinants to be included in multivariable analysis were determined based on bivariate testing (when associations had a significance of *p* < 0.05), iterative model building, and non-co-linearity. Sensitivity analysis was executed by running a model (covariates for farmers: smoking status, exposed to herbicides during last year, days per year on average mixing/applying herbicides, applied glyphosate in last 7 days before study, glove and mask use, and storage of pesticides in house; covariates for spouses: time with the windows open during the day, reported distance of home to spraying area, and herbicides drift enters houses).

### 2.6. Risk Assessment

To evaluate urinary levels of glyphosate or AMPA in this study in terms of risk assessment context, the estimated daily intake (EDI) was calculated in mg/kg BW/day for both glyphosate and AMPA, using Equation (1) [32].

EDI = (Cu × V_24_ × MW)/(FUE × BW), (1)

where Cu represents the concentration of glyphosate and AMPA in the first-morning urines, V_24_ is the total volume of urine excreted within 24 h established for adults (1.6 L/day) [33], BW corresponds to the bodyweight of each participant, FUE is the urinary excretion fraction of glyphosate and AMPA (0.01) based on [27], and MW is the molecular weight of each molecule in mg/mol.

In order to put this EDI in a risk-assessment context, a hazard quotient (HQ) was also calculated using Equation (2), with the acceptable daily intake allowance (ADI) as a reference health-based value, which is 0.5 mg/kg/d for glyphosate and AMPA [7].

HQ = EDI/ADI,(2)

If the HQ was less than 1, it was indicated that there may be a low risk for health. The HQ values of both compounds were summed in order to estimate the hazard index (HI), which estimates the increased risk due to the exposure to both compounds [34]. An HI < 1 means that the cumulative exposure to glyphosate and AMPA mixture is of little concern to public health, and HI > 1 indicates a potential health risk.

## 3. Results

### 3.1. Subsection Farmers and Spouse’s Socio-Demographic Characteristics

130 farmers and 64 spouses were recruited; among them, sufficient urine was available for 104 farmers and 50 spouses. The study also recruited 50 individuals from the general adult population. A summary of the socio-demographic characteristics of the farmers and spouses in our study population is reported in Table 1. Mean age was 37 years for farmers and 34 for spouses. Among farmers included in the study, 55.8% were married. Most farmers and spouses did not have an additional job. About one third of farmers were smokers.

### 3.2. Exposure Assessment

Exposure characteristics of the target population in this study are given in Table 2. Regarding occupational exposure to herbicides, 76.9% declared being exposed to herbicides during the last year, and 71.2% declared applying glyphosate during the previous 7 days. Half of farmers wore personal protective equipment (PPE), such as combinaison or gloves, and about one third wore masks. Half of the farmers reused their PPE. Regarding spouses, few of them reported that they had personally mixed herbicides.

### 3.3. Levels of Glyphosate and AMPA Urinary Concentrations

The descriptive statistics of the urinary glyphosate and AMPA concentrations are presented in Table 3. Glyphosate and AMPA were found in quantifiable amounts in 67% and 62% of farmers’ samples, and 56% and 48% of spouses’ samples, respectively. The mean concentrations were 1.22 μg L^−1^ for glyphosate, and 0.85 μg L^−1^ for AMPA and the P95 concentrations were 4.38 μg L^−1^ and 2.52 μg L^−1^ for glyphosate and AMPA, respectively, in the case of farmers. Regarding spouses, the mean concentrations were 0.58 μg L^−1^ for glyphosate, and 0.50 μg L^−1^ for AMPA and the P95 concentrations were 1.91 μg L^−1^ and 1.41 μg L^−1^ for glyphosate and AMPA, respectively (Table 3). For the general population, the mean values of glyphosate and AMPA were below the reported quantification limits.

### 3.4. Determinants Influencing Glyphosate and AMPA Levels

The results of binary logistic analysis of glyphosate and AMPA levels and potential determinants related to sociodemographic and occupational are presented in Table S1. According to Table S1, it can be seen that smoking status, days per year applying/mixing herbicides during the last year, applied glyphosate in the last 7 days before study, spraying glyphosate, and glove use had the strongest associations with glyphosate and AMPA levels for famers. For spouses, the strongest relationship was found between time with the windows open during the day, garden at home, reported distance of home to spraying area, and pesticides drift entering the house and glyphosate and AMPA levels.

Table 4 shows the standardized coefficients of the multiple regression model developed to assess the relationship between glyphosate and AMPA levels and the sociodemographic and occupational exposure of the target population. Glyphosate in farmers is positively associated with smoking status (Estimation coefficient: EC = 2.07; 95% CI: 1.79–2.36) and applying glyphosate in last 7 days before the study (EC = 1.23; 95% CI: 1.02–1.68). On the other hand, AMPA concentration varied significantly with days per year mixing/applying herbicides; it was higher among farmers who applied herbicides less than 7 days/year (EC = −2.68; 95% CI: −3.21–1.79) followed by those who applied it between 7 and 15 days/year (EC = −0.83; 95% CI: −1.49–0.12). Furthermore, the analysis of glyphosate and AMPA in spouses showed a significant positive relationship with the time with the windows open during the day, reported distance of home to spraying area, and pesticides drift entering the house.

### 3.5. Risk Assessment

**Figure 1:**
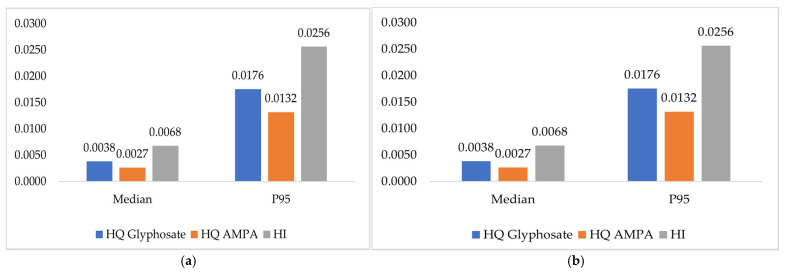
Hazard Quotient (HQ) calculated for Glyphosate and AMPA and Hazard Index (HI) at two distribution levels (Median and P95); (**a**) farmers, (**b**) spouses.

EDIs results calculated for glyphosate and AMPA for the target population are summarized in Table 5. The median EDI values obtained were 1.91 and 1.39 µg/kg BW/day for glyphosate and AMPA, respectively, for farmers, and 0.73 and 0.69 µg/kg BW/day for glyphosate and AMPA, respectively, for spouses. The highest P95 EDI was obtained for glyphosate (8.78 µg/kg BW/day, which is lower than the ADI of 0.5 mg/kg BW/day established by the EFSA. Figure 1 presents the HQ obtained independently for each analyte for farmers and spouses as well as the total HI, which is lower than 1 in both cases.

## 4. Discussion

This study provides human biomonitoring data on glyphosate and AMPA among farmers and their spouses in the Saiss Plain in Morocco. As previously discussed, studies quantifying occupational exposure to glyphosate and AMPA are very limited. There is sparse published data for glyphosate, and to the authors’ knowledge, there is no published spouse’s exposure data for AMPA. These results contribute to an area of limited data in the international literature, especially in low-and-middle income countries.

Glyphosate and AMPA were detected in around 2/3 of farmers in our study; this is similar to those reported by most previous occupational biomonitoring studies of farmers (0–75%) in Ireland and the UK [35,36], although several studies in North Carolina [37], Thailand [38] and New Zealand [39] had detection frequencies of >90%. Urinary glyphosate and AMPA concentration in our study reached maximum values of 4.60 µg L^−1^ for glyphosate and 3.75 µg L^−1^ for AMPA among farmers, and 1.91 µg L^−1^ for glyphosate and 1.63 µg L^−1^ for AMPA among spouses. Comparing the previous studies among the working population, the median of glyphosate and AMPA measured in this study among farmers was slightly lower than those found in occupationally exposed workers in eastern China [29], with values of 292 µg L^−1^ and 68 µg L^−1^, respectively (Table S2). Likewise, our reported glyphosate levels are slightly lower than the urinary glyphosate concentration of certified farmers in California and Minnesota which was <1–68 g/L 3 days post-application of glyphosate, while the value on the day of exposure was <1–233 g/L [40]. However, the mean values reported in our study were higher compared to the mean urinary glyphosate concentrations (0.21 µg L^−1^) reported in an European environmental exposure study [41]. Similar to our findings, higher detection frequencies and/or concentrations of urinary glyphosate were observed among farmers compared to nonfarmers in most other studies that quantified glyphosate in both subgroups [35,36,37].

Note that in the case of spouses, only one study is available for AMPA concentrations in an environmentally exposed area [40]. Thus, we compared our results with others’ research in the general women’s population. In the Spanish study developed by [42], DFs in lactating women were similar with those obtained in our research, indicating values of 45% for glyphosate and 60% for AMPA. In contrast, the DFs found for German women were lower by 10% and 35%, respectively [43], which are slightly lower than those measured in the current study. The maximum of AMPA obtained in our study were lower than those obtained for pregnant US women with values of 6.01 µg L^−1^. However, in the case of glyphosate, the maximum concentrations in the present study were similar to those obtained for US women (1.91 µg L^−1^) [44]. On the other hand, the P95 obtained in our study was slightly higher than those measured for lactating Spanish women with values of 0.62 µg L^−1^ for glyphosate 0.29 µg L^−1^ for AMPA [42].

Overall, comparison across studies should be interpreted with caution due to differences in analytical methods, detection sensitivities, and urine-sample type [37,45]. The high level and percentage of positive detection for both compounds in the urine of farmers and spouses could be partly explained by the season of sampling. In the present study, urine samples were collected when spraying was at its highest (May–June). Higher exposure was expected since glyphosate-based products are used more often in Morocco’s Saiss plain during this period [46]. Sampling season in relation to exposure of the general population was previously assessed by [39], who reported higher concentrations of glyphosate and AMPA in late spring/early summer than in winter. However, 71% of farmers in our study indicated using glyphosate-based products in the last seven days prior to the study, and 46% of spouses in this study lived near to the spraying area.

The study findings indicate that urinary levels of glyphosate and their metabolite AMPA in the target populations were little influenced by sociodemographic factors, while occupational activities and household-related exposures were the main determinants. Among spouses, exposure levels were more strongly influenced by factors that could modulate environmental exposure, including time with window open during the day, the presence of a garden or plants at home, reported distance of the home to spraying area, and the potential entry of herbicide drift into the house. Smoking status has been associated with higher urinary glyphosate and AMPA concentrations among farmers in the study area. Likewise, the study in Germany and US also observed that exposure to pesticide residues in the adult population is twice as high for smokers than non-smoker [47,48]. Lee et al. reported a difference between cigarette-smoking status and total mortality, depending on serum concentrations of POPs among the elderly in the U.S. [49]. However, the mechanisms behind this interaction effect are still to be identified. One possible explanation is that pesticide components may enter the body through smoking due to pesticide residues on cigarettes. Another mechanism is that smoking cigarettes can interfere with pesticide metabolism in the human body [50].

Study findings reveal that there is a potential for exposure during tasks performed in the farming area. Factors that might contribute to the exposure observed include the reuse of PPE; 90.4% of farmers reused PPE. Our results suggest that PPE use, particularly gloves, may help reduce exposure. Farmers who did not wear gloves had statistically significantly higher urinary glyphosate concentrations compared to those who wore gloves. However, the use of masks was not significant. Our result is consistent with a study conducted in USA [51], where individual contributions of inhalation following the heavy residential consumer application of a glyphosate-containing herbicide was found to be a minor route of glyphosate exposure. This could be explained by the physicochemical proprieties of glyphosate. The vapor pressure of glyphosate is extremely low. Glyphosate is usually formulated as isopropylamine salt, which has a vapor pressure of 1.6 × 10^−8^ mm Hg. Furthermore, glyphosate is soluble in water and has low affinity for organic materials such as skin [52]. Dermal penetration experiments, where glyphosate was left undistributed on skin surfaces of experimental animals and on human skin in vitro, show a percutaneous absorption of glyphosate [53]. Even if the dermal route allows a poor absorption (about 2%), it is the most commonly reported route of entry in exposed farmers [27]. Furthermore, the finding that urinary glyphosate concentrations were higher among participants who took breaks during the task than among who did not is in agreement with results reported in previous studies under similar sittings [28]. In many instances, farmers eat, drink water, and smoke during breaks without washing their hands [46].

Consistent with previous studies, handling of pesticides concentrates during mixing and loading was strongly associated with elevated pesticide exposure [54,55]. In this study, the majority of farmers performed the mixing, loading, spraying of the chemicals, and cleaning the equipment as part of the overall task they were rated for. As a result, it was not possible to assess pesticide exposure during these tasks.

Family occupational exposure to glyphosate, especially for farmers, has been considered as a predictive factor for higher exposure in their spouses. Spouses residing near to agriculture areas may be exposed to glyphosate as a result of drafting from the application areas to households’ environments where the family live. In this case, dust might have been an additional source of exposure, which can serve as a long-term reservoir of pesticide residues entering the farmers’ homes [41]. Notably, a study in the general population of California reported significantly higher glyphosate dust concentrations in households with pesticide use around the home to treat weeds, and those with nearby agricultural use of glyphosate [56]. Furthermore, a strong association was found between urinary glyphosate and time with windows open during the day. Further research quantifying glyphosate in urine and environmental dust would help to more accurately assess both short and long-term exposure to identify key determinants among family farmers [57,58].

In order to interpret the obtained levels in a risk assessment context, EDIs for glyphosate and AMPA were calculated. The obtained EDIs for farmers were higher than those recently presented by [47] for German adults (0.063 µg/kg BW/day for glyphosate and 0.057 µg/kg BW/day for AMPA). Regarding spouses, EDI95 for glyphosate are higher than those reported in a study regarding external exposure to herbicides through fruit and vegetable consumption in Spanish lactating mothers [42]. Since both HQ and HI were substantially lower than 1, Moroccan farmers and spouses under research were regarded to be at very low risk.

Overall, our research sheds light on the determinant factors affecting the concentrations of urinary glyphosate and its metabolite AMPA in low- and middle-income countries, such as Morocco. However, as further research is needed, the results should be interpreted with caution. First, the questionnaire did not include questions about what kinds of food and fruits the participants ate, and if they came from their homes’ garden. Detailed questionnaire data on dietary habits and environmental activity of participants the day prior to sampling, as well as a large sample size, would provide more information on possible exposure sources. The second limitation is the collection of only a single urine sample, which may not reflect the average or long-term exposure due to intra-individual variability, although the analysis of 24 h urine samples can be assumed to capture daily exposure to glyphosate and AMPA for one day more reliably than the first morning-void sample. Finally, height data were not collected, which prevented the calculation of body mass index (BMI) and the evaluation of its potential association with urinary levels of the toxic compounds. These considerations will be implemented in the current Moroccan human biomonitoring program (PAPOE Study), where glyphosate concentrations, along with those of other contaminants, will be determined in a population of adults, farmers, and pregnant women living in intensive agricultural areas and urban areas.

## 5. Conclusions

The assessment of potential glyphosate exposure among potentially exposed populations represents an important public health consideration, especially in light of the IARC classification of glyphosate as “probably carcinogenic to humans”. In the present research, DFs of glyphosate and AMPA are 67%, 62% for farmers, and 56% and 48% for spouses, respectively, and the median are 0.86 µg L^−1^, 0.50 µg L^−1^ for farmers, and 0.35 µg L^−1^, 0.25 µg L^−1^ for spouses, respectively. The results are in line with what was reported in published studies. Exposure determinants analysis showed some evidence that smoking status, the reuse of PPE for farmers, and reported distance of home to spraying area and herbicides drift entering houses for spouses play a role in the differences in observed glyphosate and AMAP concentrations in the individual studies. The calculated HQ showed that there were low risks due to exposure to theses herbicides among study population in the Saiss plain in Morocco. Our results contribute to improving the understanding of whole-family exposure patterns and provide information relevant for chemical regulation and policy decisions.

## Figures and Tables

**Table 1:** Basic characteristics of the study population.

	Farmers	Spouses	General Population
	*n* (%)	*n* (%)	*n* (%)
Total number	104 (100)	50 (100)	50 (100)
Age (mean)	37	34	42
<30	38 (36.5)	18 (36.0)	20 (40.0)
31–45	32 (30.8)	16 (32.0)	18 (36.0)
46–60	22 (21.2)	12 (24.0)	8 (16.0)
>61	12 (11.5)	4 (8.0)	4 (8.0)
Education			
None	52 (50.0)	22 (44.0)	2 (4.0)
Primary school	28 (26.9)	10 (20.0)	10 (20.0)
Secondary school	20 (19.2)	14 (28.0)	22 (44.0)
University degree	4 (3.8)	4 (8.0)	18 (36.0)
Marital status			
Married	58 (55.8)	48 (96.0)	42 (84.0)
Others *	46 (44.2)	2 (4.0)	8 (16.0)
Additional job			
Yes	4 (3.8)	3 (6.0)	-
No	100 (96.2)	47 (94.0)	-
Smoking status			
Smoker	43 (41.4)	0 (0)	24 (48.0)
Non–smoker	61 (58.6)	50 (100)	26 (52.0)

* Others: divorced, single, or widowed.

**Table 2:** Lifestyle and exposure to herbicides information of target population.

Lifestyle and Exposure to Herbicides	Number (%)
Farmers	
Occupational exposure	
Exposed to herbicides during last year	
Yes	80 (76.0)
No	24 (23.1)
Days per year on average mixing/applying herbicides	
<7 days	60 (57.7)
7–15 days	30 (28.8)
>15 days	14 (13.5)
Hours per day on average mixing/applying herbicides	
<2 h	34 (32.7)
3–5 h	20 (19.2)
>5 h	50 (48.1)
Applied glyphosate in last 7 days before study	
Yes	74 (71.2)
No	30 (28.8)
Participant’s herbicide and PPE use	
Glove use	
Yes	50 (48.1)
No	54 (51.9)
Mask use	
Yes	30 (28.8)
No	74 (71.2)
Coverall use	
Yes	56 (53.8)
No	48 (46.2)
Did the worker reuse personal protective equipment (PPE)?	
Yes	56 (53.8)
No	48 (46.2)
did the worker take a break during the task	
Yes	40 (38.5)
No	64 (61.5)
Which task did the workers complete?	
Collection of the pesticide from the store	
Yes	79 (75.9)
No	25 (24.1)
Mixing and loading the chemical	
Yes	90 (86.5)
No	14 (13.5)
Spraying the chemical	
Yes	90 (86.5)
No	14 (13.5)
Cleaning the equipment	
Yes	96 (92.3)
No	8 (7.7)
Storage of pesticide in house	
Yes	64 (61.5)
No	40 (38.5
Spraying glyphosate with:	
Manual knapsack	62 (59.6)
Boom sprayer	42 (40.4)
Spouses	
Spouse personally mixed any pesticides in week before study	
Yes	6 (12.0)
No	44 (88.0)
Time with the windows open during the day	
<2 h	21 (42.0)
2–4 h	14 (28.0)
>4 h	15 (30.0)
Garden or plants at home	
Yes	36 (72.0)
No	14 (28.0)
Reported distance of home to spraying area	
0–50 m	23 (46.0)
51–100 m	13 (26.0)
>100 m	14 (28.0)
Herbicide’s drift enters house	
Yes	20 (40.0)
No	30 (60.0)
Reuse of empty containers in house	
Yes	30 (60.0)
No	20 (40.0)

**Table 3:** Urinary levels of glyphosate and AMPA in target population (μg L^−1^).

	*n*	Percent Above LOD	Mean (95%CI)	Median (95%CI)	P25	P50	P75	P95	Max
Farmers									
Glyphosate	104	67	1.22 (0.91–1.60)	0.86 (0.941–1.22)	0.07	0.86	2.18	4.38	4.60
AMPA	104	61.5	0.85 (0.64–1.07)	0.50 (0.25–0.84)	0.25	0.50	1.28	2.52	3.75
Spouses									
Glyphosate	50	56	0.58 (0.41–1.24)	0.35 (0.05–0.36)	0.05	0.35	1.11	1.91	1.91
AMPA	50	48	0.50 (0.40–0.61)	0.25 (0.25–0.50)	0.25	0.25	0.63	1.41	1.63
General population									
Glyphosate	50	0	<LOQ	<LOQ	<LOQ	<LOQ	<LOQ	<LOQ	<LOQ
AMPA	50	0	<LOQ	<LOQ	<LOQ	<LOQ	<LOQ	<LOQ	<LOQ

**Table 4:** Summary of Multiple Regression Model between glyphosate and AMPA levels and various explanatory variables among farmers and spouses.

	Glyphosate (µg L^−1^)			AMPA (µg L^−1^)		
	Mean (IQR) 95%CI	Estimation Coefficient (95%CI)	*p* Value	Mean (IQR) 95%CI	Estimation Coefficient (95%CI)	*p* Value
Farmers						
Smoking status						
Smoker	2.43 (2.14–2.74)	2.07 (1.79–2.36)	**0.001**	1.08 (0.79–1.38)	0.44 (0.12–0.67)	**0.010**
Non–smoker	0.36 (0.26–0.46)			0.63 (0.48–0.81)		
Exposed to herbicides during last year						
Yes	1.51 (1.25–1.81)	0.27 (0.07–0.49)	0.053	0.91 (0.69–1.11)	0.68 (0.46–1.01)	0.064
No	0.37 (0.13–0.67)			0.49 (0.29–0.71)		
Days per year on average mixing/applying herbicides						
<7 days	0.37 (0.27–0.47)	−2.61 (−3.01–2.19)	**0.000**	0.61 (0.45–0.76)	−2.68 (−3.21–1.79)	**0.001**
7–15 days	2.15 (1.84–2.51)	−0.83 (−1.28–0.37)	**0.026**	1.16 (0.81–1.56)	−0.83 (−1.49–0.12)	**0.028**
>15 days	2.98 (2.34–3.62)			1.05 (0.66–1.47)		
Hours per day on average mixing/applying herbicides						
<2 h	0.19 (0.10–0.31)	−0.51 (−0.85–0.16)	**0.005**	0.31 (0.14–0.57)	−0.76 (−0.57–0.20)	0.320
3–5 h	1.38 (1.06–1.72)	−0.10 (−0.46–0.26)	0.592	0.96 (0.52–1.49)	−0.14 (−0.55–0.28)	0.553
>5 h	1.86 (1.47–2.21)			1.11 (0.93–1.29)		
Applied glyphosate in last 7 days before study						
Yes	1.56 (1.28–2.85)	1.23 (1.02–1.68)	**0.000**	1.04 (0.86–1.24)	0.75 (0.51–0.98)	**0.001**
No	0.37 (0.17–0.63)			0.28 (0.15–0.45)		
Glove use						
Yes	0.70 (0.44–1.02)	−1.01 (−1.45–0.55)	**0.015**	0.50 (0.33–0.68)	−0.62 (−0.92–0.32)	**0.043**
No	1.70 (1.38–2.04)			1.12 (0.89–1.36)		
Mask use						
Yes	1.03 (0.56–1.54)	−0.37 (−0.66–0.07)	0.052	0.44 (0.27–0.73)	−0.04 (−0.33–0.08)	0.847
No	1.29 (1.03–1.58)			0.97 (0.76–1.19)		
Did the worker take a break during the task						
Yes	1.61 (1.14–2.13)	0.32 (0.21–0.56)	**0.043**	0.69 (0.49–0.93)	0.06 (−0.43–0.29)	0.706
No	0.97 (0.77–1.21)			0.91 (0.70–1.13)		
Spraying glyphosate with:						
Manual knapsack	1.42 (1.13–1.72)	0.51 (0.03–0.89)	**0.040**	1.01 (0.76–1.24)	0.47 (0.15–0.79)	**0.004**
Boom sprayer	0.91 (0.59–1.62)			0.54 (1.38–0.71)		
Storage of herbicide in house						
Yes	1.49 (1.18–1.83)	0.71 (0.22–1.19)	0.140	0.85 (0.63–1.06)		
No	0.78 (0.55–1.05)			0.78 (0.55–1.05)		
Spouses						
Time with the windows open during the day						
<2 h	0.05 (0.05–0.14)	−0.62 (−1.37–0.10)	**0.010**	0.08 (0.04–0.14)	−0.28 (−0.49–0.31)	0.361
2–4 h	0.43 (0.32–0.55)	−0.63 (−1.17–0.18)	**0.031**	0.64 (0.43–0.84)	0.005 (−0.42–0.41)	0.979
>4 h	1.48 (1.23–1.71)			0.66 (0.37–10.95)		
Garden or plants at home						
Yes	0.80 (0.56–1.05)	0.64 (0.27–1.01)	0.101	0.54 (0.04–0.72)	0.40 (0.14–0.66)	0.063
No	0.16 (0.15–0.51)			0.14 (0.03–0.31)		
Reported distance of home to spraying area						
0–50 m	1.56 (1.37–1.75)	1.34 (1.15–1.57)	**0.001**	0.64 (0.34–1.00)	0.31 (0.05–0.64)	**0.037**
51–100 m	0.45 (0.31–0.40)	0.24 (0.10–0.37)	**0.004**	0.59 (0.39–0.82)	0.25 (0.05–0.46)	**0.023**
>100 m	0.10 (0.08–0.15)			0.17 (0.07–0.29)		
Herbicides drift enters house						
Yes	1.02 (0.27–1.33)	0.73 (0.48–0.98)	**0.000**	0.64 (0.44–0.84)	0.43 (0.19–0.68)	**0.001**
No	0.22 (0.10–0.35)			0.21 (0.10–0.34)		

Note: The bolded data indicate statistical significance, with *p* < 0.05.

**Table 5:** EDIs results calculated for glyphosate and AMPA (μg/kg bw/day).

Compound	EDI		Percentiles			Maximum
	GM	Median	P25	P75	P95	
Farmers						
Glyphosate	1.26	1.91	0.17	4.50	8.78	11.97
AMPA	1.39	1.39	0.65	2.92	6.60	8.22
Spouses						
Glyphosate	0.56	0.73	0.73	2.68	5.00	5.27
AMPA	0.96	0.69	0.68	1.61	3.83	4.42

## Data Availability

Data are not publicly available due to confidentiality and ethical restrictions.

## Supplementary Materials

The following supporting information can be downloaded at: https://www.mdpi.com/article/10.3390/toxics14050381/s1, Table S1: Association between glyphosate and AMPA and demographic, socio–economic, self–reported pesticide exposures and diet; Table S2: Glyphosate and AMPA levels in urine (μg L^−1^) in different studies [28,29,40,42,43,44,57,58,59].
