# Supplementary material for: Exposure of Farmers and Spouses to Glyphosate in Morocco: Urinary Levels and Predictors of Exposure
Source: Toxics. 2026 Apr 29;14(5):381. doi: 10.3390/toxics14050381 (PMC13211284; doi:10.3390/toxics14050381)
Supplement: Supplementary file 1 [file toxics-14-00381-s001.zip › toxics-4103278-supplementary.pdf]

WExposure of farmers and spouses to glyphosate in Morocco: Urinary levels and predictors of exposure

Imane Berni, Aziza Menouni, Matteo Creta, Kaoutar Chbihi, Hala Chetouani, Said Abousaid, Lode Godderis, Samir El Jaafari, Radu-Corneliu Duca

Table S1: Association between glyphosate and AMPA and demographic, socio–economic, self–reported pesticide exposures and diet.

	**Glyphosate (µg L^{-1})**				**AMPA (µg L^{-1})**			
Variable	**Mean**	**95%CI**	**Beta coefficient**	**P–Value**	**Mean**	**95%CI**	**Beta coefficient**	**P–Value**
**Farmers**								
**Age**								
< 30	0.63	0.41–0.87			0.66	0.48–0.88		
31–45	0.91	0.65–1.15	0.10	0.19	0.78	0.58–0.73	0.063	0.572
46– 60	1.33	0.85–1.83	0.129	0.063	1.14	0.65–1.02	0.134	0.056
>61	3.69	3.17–4.18	0.183	0.320	0.87	0.41–1.43	0.078	0.460
**Education**								
None	1.70	1.36–2.15						
Primary school	1.02	0.68–1.40	–0.042	0.113	0.85	0.68–1.03	0.089	0.389
Secondary school	0.36	0.19–0.56	–0.122	0.071	1.01	0.61–1.46	–0.096	0.351
University	0.58	0.00–1.16	–0.173	0.063	0.65	0.34–0.98	–0.198	0.052
**Marital status**			–0.291	0.302	0	0	–0.151	0.126
Married	1.54	1.15–1.91			0.93	0.73–1.15		
Others	0.81	0.61–1.03			0.68	0.46–0.91		
**Smoking status**			0.399	0.043			0.134	0.049
Smoker	2.43	2.14–2.74			1.08	0.79–1.38		
Non–smoker	0.36	0.26–0.46			0.63	0.48–0.81		
**Exposed to herbicides during last year**			0.379	0.000			0.217	0.027
Yes	1.51	1.25–1.81			0.91	0.69–1.11		
No	0.37	0.13–0.67			0.49	0.29–0.71		
**Days per year on average mixing/applying herbicides**								
< 7 days	0.37	0.27–0.47			0.61	0.45–0.76		
7–15 days	2.15	1.84–2.51	0.628	0.000	1.16	0.81–1.56	0.291	0.004

> 15 days	2.98	2.34–3.63	0.714	0.000	1.05	0.66–1.47	0.180	0.069
**Hours per day on average mixing/applying herbicides**								
< 2h	0.19	0.10–0.31			0.31	0.14–0.57		
3–5 h	1.38	1.06–1.72	0.227	0.000	0.96	0.52–1.49	0.455	0.056
>5h	1.86	1.47–2.21	0.240	0.043	1.11	0.93–1.29	0.070	0.462
**Applied glyphosate in last 7 days before study**			0.432	0.000			0.415	0.000
Yes	1.56	1.28–1.85			1.04	0.86–1.24		
No	0.37	0.17–0.63			0.28	0.15–0.45		
**Participants herbicide & PPE use**								
**Glove use**			–0.401	0.000			–0.374	0.000
Yes	0.70	0.44–1.02			0.50	0.33–0.68		
No	1.70	1.38–2.04			1.12	0.89–1.36		
**Mask use**			–0.098	0.032			–0.290	0.003
Yes	1.03	0.56–1.54			0.44	0.27–0.73		
No	1.29	1.03–1.58			0.97	0.76–1.19		
**Comnibainson use**			–0.126	0.202			–0.054	0.589
Yes	1.07	0.78–1.39			0.78	0.58–0.96		
No	1.39	1.02–1.76			0.86	0.63–1.13		
**did the worker take a break during the task**			0.25	0.011			–0.128	0.194
Yes	1.61	1.14–2.13			0.69	0.49–0.93		
No	0.97	0.77–1.21			0.91	0.70–1.13		
**Sprying glyphosate with:**			0.202	0.040			0.279	0.004
Manual knapsack	1.42	1.13–1.72			1.01	0.76–1.24		
Boom sprayer	0.91	0.59–1.26			0.54	1.38–0.71		
**Which task did the workers complete?**								
**Collection of the herbicide from the store**			–0.036	0.719			–0.003	0.973

Yes	1.19	0.89–1.47			0.81	0.64–1.01		
No	1.30	0.84–1.82			0.83	0.58–1.10		
**Stotage of pherbicide in house**			0.276	0.005			0.034	0.732
Yes	1.49	1.18–1.83			0.85	0.63–1.06		
No	0.78	0.55–1.05			0.78	0.55–1.05		
**Spouses**								
**Age** (mean)								
< 30	0.72	0.41–1.05			0.38	0.18–0.57		
31–45	0.21	0.04–0.42	–0.062	0.552	0.29	0.15–0.44	–0.078	0.454
46– 60	0.73	0.35–1.13	–0.042	0.685	0.38	0.10–0.74	0.066	0.523
>61	1.09	0.31–1.87	0.176	0.082	1.16	0.08–1.24	0.127	0.208
**Education**								
None	0.62	0.32–0.90			0.54	0.30–0.78		
Primary school	0.70	0.27–1.21	–0,041	0.691	0.46	0.26–0.65	–0.068	0.632
Secondary school	0.28	0.07–0.74	0.010	0.925	0.08	0.10–0.16	–0.448	0.065
University degree	0.87	0.64–1.10	–0.010	0.924	0.72	0.25–1.18	0.10	0.465
**Time with the windows open during the day**								
< 2h	0.05	0.00–0.14			0.08	0.04–0.14		
2 – 4h	0.43	0.32–0.55	0.255	0.001	0.64	0.43–0.84	0.533	0.000
> 4h	1.48	1.23–1.71	0.973	0.000	0.66	0.37–0.95	0.560	0.004
**Garden or plants at home**			0.286	0.021			0.123	0.030
Yes	0.80	0.56–1.05			0.54	0.40–0.72		
No	0.16	0.15–0.51			0.14	0.03–0.31		
**Reported distance of home to spraying area**								
0–50m	1.56	1.37–1.75			0.64	0.34–1.00		
51–100m	0.45	0.31–0.40	0.245	0.000	0.59	0.39–0.82	0.403	0.005
>100m	0.10	0.08–0.15	0.991	0.000	0.17	0.07–0.29	0.467	0.001

**Herbicides drift enters house**			0.502	0.000			0.119	0.004
Yes	1.02	0.27–1.33			0.64	0.44–0.84		
No	0.22	0.10–0.35			0.21	0.10–0.34		
**Reuse of empty containers in house**			–0.006	0.959			0.126	0.486
Yes	0.64	0.39–0.91			0.41	0.24–0.60		
No	0.51	0.26–0.77			0.41	0.21–0.65		

Table S2. Glyphosate and AMPA levels in urine (µg L–1) in different studies.

Reference	Type of study	Country	Analytical method	participants number	Glyphosate urine cc (µg L^{-1})				AMPA urine cc (µg L–^{1})			
					LOD/LOQ	Statistic & value	Max	Range	LOD/LOQ	Statistic & value	Max	Range
Ruiz et al., 2021	Lactating women	Spain	LC–MS/MS	97	OLD 0.1	GM 0.25	1.31		0.1	0.27	2.38	
Lesseur et al., 2021	Pregnant women	US	LC–MS/MS	94	LOD 0.041	GM 0.38	1.9		LOD 0.04	GM 0.40	6.01	
Zhang et al., 2020	Pesticide production plants	China	GC–MS/MS	End of work shift samples. N=134	LOD 20	GM 262 Median 292	17,202	<0.020–17.202	10	GM 72 Median 68	2730	<0.010–2.730
Perry et al., 2019	US agricultural cohort study	United States	LC–MS/MS	18 farmers—8 hrs after application and 17 no–applicators	LOD 0.4	Median < LOD	12.0	1.3–12.0	1	Median <LOD	NR	
Connolly et al., 2017	Horticulture amenity gardening	Ireland	LC–MS/MS	17 workers—31 paired samples	LOQ 0.6	GM 0.66 AM 1.35	10.66		N/A	N/A	N/A	N/A
Conrad et al., 2017	Environmental exposures	Germany	GC–MS/MS	399 samples adultes	LOQ 0.1	Median < LOQ	2,8			Median < LOQ	1,88	
Mesnage et al., 2013	Farm family exposure study	France	HPLC–ESI–MS	1 farmer, spouse	LOD 1 LOQ 2	Overall results not given	9.5		NR	ND	ND	

Jayasumana et al., 2015	Investigate glyphosate levels in Sri Lankan Agricultural Nephropathy (SAN) patients	Sri Lanka	ELISA	30–3 groups of 10).		Median Group 1: 56.8 Group 2: 73.5 Group 3: 3.3	≥80				
Acquavella et al., 2004	Occupational and residential exposures in agricultural setting.	United States	HPLC following ion exchange	48 farmers, 48 spouses & 79 children.	LOD 1	GM Farmers 3.2 Spouse 6.4	Farers 233 Spouse 3	< 1–233/ <1–2	N/A	N/A	N/A

References

Acquavella, J.F., Alexander, B.H., Mandel, J.S., Gustin, C., Baker, B., Chapman, P., Bleeke, M., 2004. Glyphosate biomonitoring for farmers and their families: results from the Farm Family Exposure Study. Environ Health Perspect 112, 321–326.

Connolly, A., Jones, K., Galea, K.S., Basinas, I., Kenny, L., McGowan, P., Coggins, M., 2017. Exposure assessment using human biomonitoring for glyphosate and fluroxypyr users in amenity horticulture. International Journal of Hygiene and Environmental Health 220, 1064–1073. https://doi.org/10.1016/j.ijheh.2017.06.008

Conrad, A., Schröter-Kermani, C., Hoppe, H.-W., Rüther, M., Pieper, S., Kolossa-Gehring, M., 2017. Glyphosate in German adults – Time trend (2001 to 2015) of human exposure to a widely used herbicide. International Journal of Hygiene and Environmental Health 220, 8–16. https://doi.org/10.1016/j.ijheh.2016.09.016

Jayasumana, C., Gunatilake, S., Siribaddana, S., 2015. Simultaneous exposure to multiple heavy metals and glyphosate may contribute to Sri Lankan agricultural nephropathy. BMC Nephrol 16, 103. https://doi.org/10.1186/s12882-015-0109-2

Lesseur, C., Pirrotte, P., Pathak, K.V., Manservisi, F., Mandrioli, D., Belpoggi, F., Panzacchi, S., Li, Q., Barrett, E.S., Nguyen, R.H.N., Sathyanarayana, S., Swan, S.H., Chen, J., 2021. Maternal urinary levels of glyphosate during pregnancy and anogenital distance in newborns in a US multicenter pregnancy cohort. Environmental Pollution 280, 117002. https://doi.org/10.1016/j.envpol.2021.117002

Mesnage, R., Bernay, B., Séralini, G.-E., 2013. Ethoxylated adjuvants of glyphosate-based herbicides are active principles of human cell toxicity. Toxicology 313, 122–128. https://doi.org/10.1016/j.tox.2012.09.006

Perry, M.J., Mandrioli, D., Belpoggi, F., Manservisi, F., Panzacchi, S., Irwin, C., 2019. Historical evidence of glyphosate exposure from a US agricultural cohort. Environ Health 18, 42. https://doi.org/10.1186/s12940-019-0474-6

Ruiz, P., Dualde, P., Coscollà, C., Fernández, S.F., Carbonell, E., Yusà, V., 2021. Biomonitoring of glyphosate and AMPA in the urine of Spanish lactating mothers. Science of The Total Environment 801, 149688. https://doi.org/10.1016/j.scitotenv.2021.149688

Zhang, F., Xu, Y., Liu, X., Pan, L., Ding, E., Dou, J., Zhu, B., 2020. Concentration Distribution and Analysis of Urinary Glyphosate and Its Metabolites in Occupationally Exposed Workers in Eastern China. Int J Environ Res Public Health 17, E2943. https://doi.org/10.3390/ijerph17082943
